# The Brazilian version of the Bournemouth questionnaire for low back pain: translation and cultural adaptation

**DOI:** 10.1590/1516-3180.2018.0482120419

**Published:** 2019-08-08

**Authors:** Danilo Harudy Kamonseki, Carlos Luques Fonseca, Letícia Bojikian Calixtre

**Affiliations:** I MSc. Doctoral Student, Department of Physiotherapy, Universidade Federal de São Carlos (UFSCar), São Carlos (SP), Brazil; II PT. Professor, Faculdade Anhanguera, Sorocaba (SP), Brazil; III PhD. Professor, Department of Physiotherapy, Universidade Federal de São Carlos (UFSCar), São Carlos (SP), Brazil.

**Keywords:** Translations, Surveys and questionnaires, Outcome assessment, Back pain

## Abstract

**BACKGROUND:**

The Bournemouth questionnaire is a multidimensional instrument for evaluating health domains among patients with low back pain.

**OBJECTIVE:**

The objective of this study was to translate and cross-culturally adapt the Bournemouth questionnaire for individuals with low back pain, to Brazilian Portuguese.

**DESIGN AND SETTINGS:**

This was a cross-sectional study conducted at the Federal University of São Carlos.

**METHODS:**

The Brazilian version of the Bournemouth questionnaire was developed following the processes of translation, back-translation, committee review and pre-testing. The translation phase involved two independent bilingual translators whose mother language was Brazilian Portuguese. The back-translation phase involved two independent translators whose mother language was English. In order to verify comprehension of the questionnaire, 44 individuals (43.1% men) with low back pain, and with mean age of 45.4 ± 13.8 years, participated in the pre-testing phase.

**RESULTS:**

During the translation phase, some terms and expressions were changed to obtain cultural equivalence to the original Bournemouth questionnaire. In the pre-testing phase, each item of the questionnaire showed a comprehension level of over 90%.

**CONCLUSION:**

The Bournemouth questionnaire was translated and culturally adapted to the Portuguese language, to be used among individuals with low back pain.

## INTRODUCTION

Low back pain is one of the most common health conditions worldwide. The Global Burden of Disease study showed that low back pain was the leading cause of the overall number of years lived with diseases in 188 countries from 1990 to 2013.^1^ The results relating to the burden of disease in Brazil from 1990 to 2016 showed that low back pain was a main cause of disease.^2^ In 2015, the overall point-prevalence of activity-limiting low back pain was 7.3%, thus implying that 540 million people were affected at any one time.^3^

The one-year incidence of low back pain has been found to range from 6.3% to 15.4% for the first episode, and from 1.5% to 36% for any episode of low back pain. The prevalence is higher among females and people aged 40 to 80 years.^4^^,^^5^ Low back pain is associated with high costs, and the estimated indirect costs in United States have been found to be 19 billion dollars per year.^6^

Analysis on any disease or injury requires standardized tools that measure patient conditions with precision and quality, in order to follow the clinical course and progression of rehabilitation and to verify treatment efficacy in relation to self-perceived health. Questionnaires and functional scales are important for clinical practice and scientific research, since they can measure subjective information in an efficient, trustworthy and low-cost manner.^7^^,^^8^^,^^9^ Questionnaires created in other languages have to be adapted to the environment in which they will be used, considering the language and culture. Therefore, the process of translation and cross-cultural adaptation of a questionnaire needs to be standardized to reach equivalence between the original and the translated versions. Subsequently, the psychometric properties of the questionnaire need to be evaluated to ensure that the tool possesses characteristics, validity and reliability that are similar to those of the original version.^9^^,^^10^^,^^11^

Low back pain is a condition of complex and subjective nature. It is more than just a response to a nociceptive stimulus to a tissue lesion: it is also a multidimensional experience described by the biopsychosocial model, which includes pain-related, disability-related, cognitive and affective domains.^5^^,^^8^^,^^12^

The Bournemouth questionnaire was created by Bolton and Breen in 1999,^8^ to fill the need for a tool that was able to measure multidimensional health domains, such as pain, function, incapacity and psychological and social factors among patients with low back pain. This questionnaire can be easily applied and is reproducible and responsive to clinical alterations, which makes it appropriate for use in scientific research and clinical practice, for monitoring the progression of symptoms and for assisting in planning treatments for patients with low back pain.^8^^,^^12^^,^^13^ Furthermore, the Bournemouth questionnaire has been linked to many important core sets contained within the International Classification of Functioning (ICF), Disability and Health, such as body function, activities and participation.^12^

The original version of this questionnaire was written in English, but it has already been translated and culturally adapted to different languages such as German,^14^ Danish^15^ and Turkish^16^ and has been widely used as an evaluation tool in several studies.^17^^,^^18^^,^^19^ The neck pain version of the Bournemouth questionnaire has already been translated into Brazilian Portuguese.^20^ However, the low back pain version has not been translated to Brazilian Portuguese yet. For this to be used in Brazil, it needs to be translated and culturally adapted.

## OBJECTIVES

The purpose of this study was to translate and culturally adapt the Bournemouth questionnaire for low back pain, to Brazilian Portuguese.

## METHODS

### Translation and adaptation procedures

**Figure 1.**
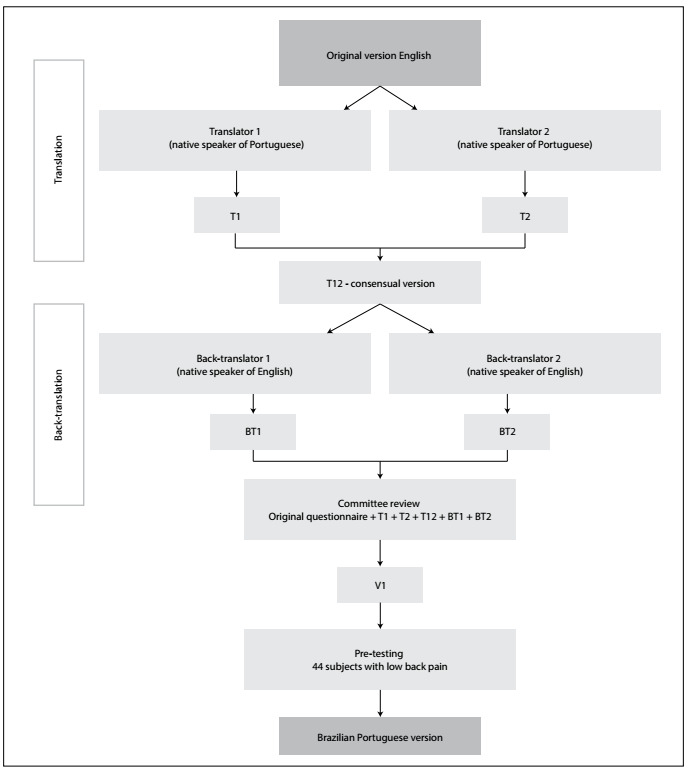
Study flowchart.

The author of the original Bournemouth questionnaire confirmed the originality of this study. The translation and cultural adaptation procedures were based on previous studies^20^^,^^21^^,^^22^^,^^23^ and followed the proper guidelines.^10^^,^^11^^,^^24^ The procedures were divided into the following stages: translation, back-translation, expert committee review and pre-testing (Figure 1).

The original version was translated from English to Portuguese by two independent bilingual translators whose mother language was Brazilian Portuguese. One of them was aware of the constructs of the questionnaire, while the other one was a layman regarding this subject. In the next phase, the translated versions that had been elaborated independently (T1 and T2) were compared and discussed by the committee, which was composed of three specialized physical therapists and both of the bilingual translators who had participated in the previous phase. In the event of any disagreement, alterations were made to the consensual Portuguese version (T12), while maintaining the main characteristics of the original questionnaire.

**Figure 2.**
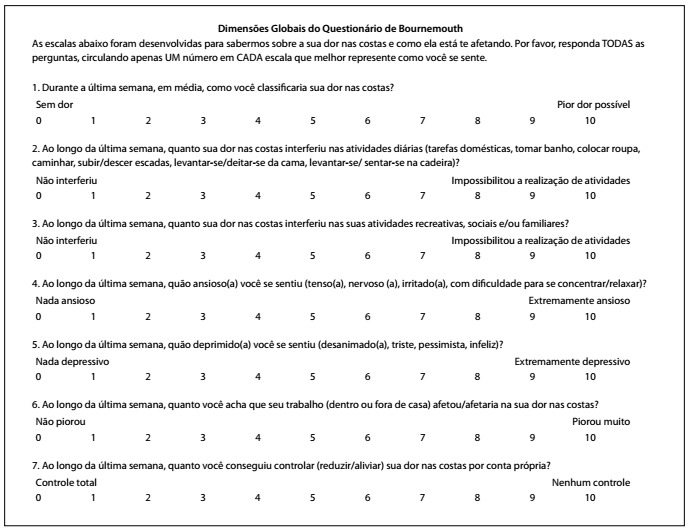
Brazilian Portuguese version of the Bournemouth questionnaire for low back pain.

T12 was translated back to English by two independent translators whose mother language was English. These translators did not have access to the original questionnaire. They generated two new versions: BT1 and BT2. Subsequently, the same members of the previous committee participated in a second meeting to verify the differences among the translated versions (T1, T2, T12, BT1 and BT2), in relation to the original questionnaire. They verified semantics and idiomatic and cultural equivalence, and they modified or eliminated any irrelevant, inadequate or ambiguous topics. The second meeting resulted in a pre-final version (V1), which was then applied in the pre-testing stage (Figure 2).

After the translation procedures had been completed, 44 subjects older than 18 years, who were recruited through verbal and digital invitation, took part in the cultural adaptation part of the study. Participants were considered eligible for the study if they had low back pain and were physically and mentally able to give responses to the questionnaires.

The pre-testing stage was performed to verify the comprehension and acceptability of the questions and answers among patients with low back pain. These subjects were asked to read and answer the questionnaire. Then, the researcher asked whether they comprehended the questions, what they understood and whether they had any suggestion for modifying the questions, in the event that any topic remained unanswered.^20^^,^^21^^,^^22^^,^^23^

This study was approved by the Human Research Ethics Committee (approved on September 9, 2014, under protocol no. 31477314.0.0000.5512). All participants received verbal and written explanations about the aims and methodology of the study, and those who agreed to participate signed an informed consent agreement.

### Score calculation

The original Bournemouth questionnaire comprises seven questions, and each of them represents a different dimension of low back pain: pain intensity (question 1), functional status in daily living (question 2), functional status in social activities (question 3), affective dimensions of anxiety (question 4), affective dimensions of depression (question 5), cognitive aspects of fear-avoidance behavior (question 6) and pain locus of control (question 7).^8^^,^^15^ Each topic of the Bournemouth questionnaire is scored using an 11-point numerical rating scale. The final total score ranges from 0 to 70 and is obtained by summing the scores of the seven topics. Higher scores reflect greater pain and disability.^8^^,^^15^

## RESULTS

The results from the translation stage (T1, T2 and T12) are described in Table 1. The results from the back-translation phase (BT1 and BT2) are described in Table 2 and the V1 version of the questionnaire is shown in Figure 2.

**Table 1.** Translation and modification of the consensual version

Bournemouth questionnaire	T1 and T2 versions	T12 consensual version
1. Over the past week, on average how would you rate your back pain? No pain/Worst pain possible	T1. Ao longo da última semana, como você classificaria, em média, sua dor nas costas? Não senti dor/Senti a pior dor possível. T2. Durante a última semana, em média, como você classificaria sua dor nas costas? Sem dor/Pior dor possível.	Durante a última semana, em média, como você classificaria sua dor nas costas? Sem dor/Pior dor possível.
2. Over the past week, how much has your back pain interfered with your daily activities (housework, washing, dressing, walking, climbing stairs, getting in/out of bed/chair)? No interference/Unable to carry out activities	T1. Ao longo da última semana, quanto sua dor nas costas interferiu nas atividades cotidianas (trabalhos do lar, banhar-se, vestir-se, caminhar, subir/descer escadas, levantar-se/deitar-se na cama, levantar-se/sentar-se na cadeira)? Não houve interferência/Sequer consegui executar essas atividades. T2. Durante a última semana, quanto sua dor nas costas interferiu suas atividades diárias (tarefas domésticas, limpeza, vestir-se, andar, subir escadas, deitar-se ou levantar-se da cama/de cadeiras)? Não interferiu/Impossibilitou a realização de atividades.	Ao longo da última semana, quanto sua dor nas costas interferiu nas atividades diárias (tarefas domésticas, tomar banho, colocar roupa, caminhar, subir/descer escadas, levantar-se/deitar-se na cama, levantar-se/sentar-se na cadeira)? Não houve interferência/Impossibilitou a realização de atividades.
3. Over the past week, how much has your back pain interfered with your ability to take part in recreational, social, and family activities? No interference/Unable to carry out activities	T1. Ao longo da última semana, quanto sua dor nas costas interferiu na sua aptidão para participar de atividades recreativas, sociais e/ou familiares? Não houve interferência/Sequer consegui executar essas atividades. T2. Durante a última semana, quanto sua dor nas costas interferiu na sua habilidade em participar em atividades recreativas, sociais e familiares? Não interferiu/Impossibilitou a realização de atividades.	Ao longo da última semana, quanto sua dor nas costas interferiu nas suas atividades recreativas, sociais e/ou familiares? Não interferiu/Impossibilitou a realização de atividades.
4. Over the past week, how anxious (tense, uptight, irritable, difficulty in concentrating/relaxing) have you been feeling? Not at all anxious/Extremely anxious	T1. Ao longo da última semana, quão ansioso(a) você se sentiu? (tenso(a), irritadiço(a), com dificuldade para se concentrar/relaxar). Não me senti ansioso(a)/Me senti extremamente ansioso(a). T2. Durante a última semana, quão ansioso (tenso, nervoso, irritado, com dificuldade em se concentrar/relaxar) você tem se sentido? Nada ansioso/Extremamente ansioso.	Ao longo da última semana, quão ansioso(a) você se sentiu (tenso(a), irritado(a), com dificuldade para se concentrar/relaxar)? Nada ansioso/Extremamente ansioso.
5. Over the past week, how depressed (down-in-the-dumps, sad, in low spirits, pessimistic, unhappy) have you been feeling? Not at all depressed/Extremely depressed	T1. Ao longo da última semana, quão deprimido(a) você se sentiu? (desanimado(a), triste, pessimista, infeliz). Não me senti deprimido(a)/Me senti extremamente deprimido(a). T2. Durante a última semana, quão depressivo (pra baixo, triste, desanimado, pessimista, infeliz) você tem se sentido? Nada depressivo/Extremamente depressivo.	Ao longo da última semana, quão deprimido(a) você se sentiu (desanimado(a), triste, pessimista, infeliz)? Nada depressivo/Extremamente depressivo.
6. Over the past week, how have you felt your work (both inside and outside the home) has affected (or would affect) your back pain? Have made it no worse/Have made it much worse	T1. Ao longo da última semana, quanto você acha que seu trabalho (dentro ou fora de casa) interferiu na sua dor nas costas? Não interferiu nada/Piorou muito a dor nas costas. T2. Durante a última semana, quanto você sentiu que seu trabalho (tanto dentro quanto fora de casa) afetou (ou afetaria) sua dor nas costas? Não piorou/Piorou bastante.	Ao longo da última semana, quanto você acha que seu trabalho (dentro ou fora de casa) afetou na sua dor nas costas? Não piorou/Piorou muito.
7. Over the past week, how much have you been able to control (reduce/help) your back pain on your own? Completely control it/No control whatsoever	T1. Ao longo da última semana, quanto você conseguiu controlar (reduzir/aliviar) sua dor nas costas por conta própria? Consegui controlar completamente/Não consegui controlar nem um pouco. Consegui controlar completamente/Não consegui controlar nem um pouco. T2. Durante a última semana, quanto você tem sido capaz de controlar (reduzir/ajudar) sozinho sua dor nas costas? Controle total/Nenhum controle.	Ao longo da última semana, quanto você conseguiu controlar (reduzir/aliviar) sua dor nas costas por conta própria? Controle total/Não consegui controlar.

**Table 2.** Back-translation phase. Differences between BT1 and BT2 and the original version

Bournemouth questionnaire	Differences between BT1 and BT2 versions
1. Over the past week, on average how would you rate your back pain? No pain/Worst pain possible	BT1. During the last week, on average, how would you classify your back pain? Without pain/Worst pain possible. BT2. During the past week, on average, how would you rate your back pain? No pain/Worst pain possible.
2. Over the past week, how much has your back pain interfered with your daily activities (housework, washing, dressing, walking, climbing stairs, getting in/out of bed/chair)? No interference/Unable to carry out activities	BT1. Over the last week, how much did your back pain interfere in daily activities (housework, bathing, dressing, walking, going up/down stairs, getting up from/sitting down on a chair)? Did not interfere/Made performance of activities impossible. BT2. Over the past week, to what extent did your back pain interfere in your daily activities (household chores, washing, dressing, walking, going up/downstairs, getting into/out of bed, getting up from/sitting down on a chair)? It did not interfere/Unable to carry out activities.
3. Over the past week, how much has your back pain interfered with your ability to take part in recreational, social, and family activities? No interference/Unable to carry out activities	BT1. Over the last week, how much did your back pain interfere with your recreational, social and/or family activities? Did not interfere/Made performance of activities impossible. BT2. Over the past week, to what extent did your back pain interfere in your recreational, social and/or family activities? It did not interfere/Unable to carry out activities.
4. Over the past week, how anxious (tense, uptight, irritable, difficulty in concentrating/relaxing) have you been feeling? Not at all anxious/Extremely anxious	BT1. Over the last week, how anxious have you felt (tense, irritated, with difficulty concentrating/relaxing)? Not anxious at all/Extremely anxious. BT2. Over the past week, how anxious have you felt (tense, irritated, unable to concentrate/relax)? Not at all anxious/Extremely anxious.
5. Over the past week, how depressed (down-in-the-dumps, sad, in low spirits, pessimistic, unhappy) have you been feeling? Not at all depressed/Extremely depressed	BT1. Over the last week, how depressed have you felt (gloomy, sad, pessimistic, unhappy)? Not depressed at all/Extremely depressed. BT2. Over the past week, how depressed have you felt (down, sad, pessimistic, unhappy)? Not at all depressed/Extremely depressed.
6. Over the past week, how have you felt your work (both inside and outside the home) has affected (or would affect) your back pain? Have made it no worse/Have made it much worse	BT1. Over the last week, to what extent do you think your work (inside or outside the home) affected/would affect your back pain? Did not get worse/Got much worse. BT2. Over the past week, to what extent do you think that your work (inside or outside of your home) affected/could affect your back pain? It did not make it worse/It made it a lot worse.
7. Over the past week, how much have you been able to control (reduce/help) your back pain on your own? Completely control it/No control whatsoever	BT1. Over the last week, to what extent have you been able to control (reduce/relieve) your back pain on your own? Total control/No control. BT2. Over the last week, to what extent were you able to reduce/alleviate your back pain by yourself? Completely/Not at all.

BT1 = back-translation 1; BT2 = back-translation 2.

In the pre-testing phase, 44 individuals (43.1% men) answered the pre-testing version of the Bournemouth questionnaire, in Brazilian Portuguese (V1). The participants were native speakers of Portuguese with mean age of 45.4 ± 13.8 years, who had presented low back pain for 70.8 ± 143.6 months. Ten individuals (22.7%) had not completed elementary school and 10 (22.7%) had completed it; 14 (31.81%) had completed high school and 10 (22.7%) had a bachelor’s degree. In this phase, the subjects did not have any suggestions for improving the comprehension of the topics. According to these individuals, there was no difficulty in filling out the questionnaire. All questions showed comprehension level higher than 90%, and therefore it was not necessary to modify V1 after the pre-testing phase.

## DISCUSSION

The purpose of this study was to translate and culturally adapt the Bournemouth questionnaire for low back pain, to Brazilian Portuguese. Translation and cultural adaptation studies make it possible to provide common measurements for investigations within different cultural contexts, a standard measurement for application in international studies and a means for comparisons between national/cultural groups. Moreover, they have the advantage of being less costly and less time-consuming than it would be to generate a new measurement.^10^^,^^11^^,^^24^

To ensure equivalence between the original questionnaire and the new adapted version, and to maintain the characteristics of the original instrument at a conceptual level across different cultures, the methods used in cross-culturally adapting a questionnaire should follow the guidelines proposed by Beaton in 2000.^10^ The translation and cross-cultural adaptation methods used in the present study have become well established in the literature and have been applied in several studies.^20^^,^^21^^,^^22^^,^^23^ Versions of the Bournemouth questionnaire translated to other languages have been provided through previous studies^14^^,^^15^^,^^16^ using the same methodology. The cross-cultural adaptation process was successfully completed in all cases.

In the translation phase for producing the Portuguese version of the Bournemouth questionnaire, the T12 consensual version was elaborated in order to avoid ambiguous or hard-to-comprehend words such as *cotidianas, sequer, aptidão* and *irritadiço*, which were present in at least one of the translations. In the back-translation phase, there was no difference in meanings between the translation and the original version, thus indicating that the adaptations that were made in the initial phase did not alter the meanings of the topics. In the pre-testing phase, the level of comprehension of all the topics was higher than 90%, which indicates that the new version of this questionnaire was easily understood.

Although the guidelines (2000)^10^ highly recommend that, after the translation and adaptation process, the reliability and construct validity of the product should be verified, several studies have reported the translation and cross-cultural adaptation phases without analysis on the psychometric properties.^22^^,^^23^^,^^25^^,^^26^^,^^27^ It is valuable to report a detailed description of the translation and cultural adaptation process before the validation, in order to prevent occurrences of multiple translations of the same tool and to avoid the extensive amount of work that would be entailed in translating and/or validating the same tool more than once.

The test-retest reliability of other versions of the Bournemouth questionnaire has been analyzed, and this analysis showed that the questionnaire had excellent reliability.^8^^,^^14^^,^^15^^,^^16^ This questionnaire has also been shown to have good internal consistency, with the capacity to demonstrate clinically significant improvement in patients’ conditions.^8^^,^^14^^,^^15^^,^^16^^,^^17^^,^^28^ The psychometric properties of the Brazilian Portuguese version are currently under evaluation and the results from this assessment will soon be available in the literature. This process will allow its use in an appropriate manner in Brazil.

## CONCLUSION

The Bournemouth questionnaire was translated and culturally adapted to Brazilian Portuguese, in a comprehensive version for evaluating low back pain among Brazilians.
